# Patient‐Focused Drug Development Methods for Benefit–Risk Assessments: A Case Study Using a Discrete Choice Experiment for Antiepileptic Drugs

**DOI:** 10.1002/cpt.1231

**Published:** 2018-10-25

**Authors:** Emily A.F. Holmes, Catrin Plumpton, Gus A. Baker, Ann Jacoby, Adele Ring, Paula Williamson, Anthony Marson, Dyfrig A. Hughes

**Affiliations:** ^1^ Centre for Health Economics and Medicines Evaluation Bangor University Bangor UK; ^2^ Department of Molecular and Clinical Pharmacology University of Liverpool Liverpool UK; ^3^ Department of Public Health and Policy University of Liverpool Liverpool UK; ^4^ Institute of Psychology, Health and Society University of Liverpool Liverpool UK; ^5^ Medical Research Council North West Hub for Trials Methodology Research Department of Biostatistics University of Liverpool Liverpool UK; ^6^ Walton Centre National Health Service Foundation Trust Liverpool UK

## Abstract

Regulatory decisions may be enhanced by incorporating patient preferences for drug benefit and harms. This study demonstrates a method of weighting clinical evidence by patients’ benefit–risk preferences. Preference weights, derived from discrete choice experiments, were applied to clinical trial data to estimate the expected utility of alternative drugs. In a case study, the rank ordering of antiepileptic drugs (AEDs), as indicated from clinical studies, was compared with ordering based on weighting clinical evidence by patients’ preferences. A statistically significant change in rank ordering of AEDs was observed for women of childbearing potential who were prescribed monotherapy for generalized or unclassified epilepsy. Rank ordering inferred from trial data, valproate > topiramate > lamotrigine, was reversed. Modeling the expected utility of drugs might address the need to use more systematic, methodologically sound approaches to collect patient input that can further inform regulatory decision making.

**Study Highlights**

**WHAT IS THE CURRENT KNOWLEDGE ON THE TOPIC?**

Discrete choice experiments have been recognized as a suitable method for eliciting evidence on patients’ preferences to inform regulatory benefit–risk assessments.

**WHAT QUESTION DID THIS STUDY ADDRESS?**

What is the impact of weighting clinical evidence by patients’ benefit–risk preferences, and how can this be achieved in practice?

**WHAT DOES THIS STUDY ADD TO OUR KNOWLEDGE?**

On the basis of a case study of antiepileptic drugs (AEDs), patients were willing to accept a reduction in the chance of seizure remission in exchange for a reduction in the risk of adverse effects. This resulted in changes to the trial‐based rank ordering of AEDs, determined from time to treatment failure.

**HOW MIGHT THIS CHANGE CLINICAL PHARMACOLOGY OR TRANSLATIONAL SCIENCE?**

Explicit consideration of patient preferences could lead to different conclusions relating to the benefit–risk profiles of medicines. This has implications for drug development and regulation, in how patient values and clinical attributes may be integrated to inform benefit–risk assessments.

Drug development and regulatory decision making require explicit evaluation of benefits and risks. In balancing the benefits and risks of drugs, judgements are required on the maximum acceptable risk (MAR) of harm for an expected health benefit. Traditionally, these judgements have relied on expert clinical opinion of the available evidence. More recently, however, there is increasing acknowledgment that patients’ preferences with respect to trade‐offs in harms for benefits may differ from those of clinical experts and that patient perspectives need to be considered in benefit–risk assessments.^1^, ^2^

The US Food and Drug Administration (FDA) Center for Drug Evaluation and Research currently implements a qualitative approach via the Patient‐Focused Drug Development Program under the fifth and sixth authorizations of the Prescription Drug User Fee Act. This involves patients in the approvals process by convening public meetings to discuss the impact of disease on patients’ daily lives and patients’ perspectives on treatment benefits and adequacy.^3^ The European Medicines Agency's Committee for Medicinal Products for Human Use piloted patient involvement in benefit–risk discussions through their participation in expert group meetings and the scientific advice/protocol assistance procedure.^4^ The Committee for Medicinal Products for Human Use continues to involve patients in oral explanations when it is believed this could be of benefit. The FDA's Center for Devices and Radiological Health (CDRH), by contrast, has adopted a more quantitative approach and considers evidence relating to patients’ perspectives of what constitutes meaningful benefit–risk.^2^, ^5^

Moving forward, the 21^st^ Century Cures Act (section 3002) requires the FDA to develop guidance (by quarter 4 2021) for integrating relevant patient experience data in benefit–risk assessments for new drugs and biological agents.^6^ Although specific details are currently unknown, it is worth noting the CDRH's guidance includes 11 recommendations for the conduct of quantitative patient preference studies,^5^, ^7^ and a project coordinated by the European Medicines Agency outlined a range of methods.^8^ Both organizations identify discrete choice experiments (DCEs) among potential options for use in benefit–risk assessments of medicines.

Stated preference DCEs are a method for quantifying the relative importance of different treatment characteristics (attributes), trade‐offs between these attributes, and respondents’ total satisfaction (utility) with specified treatments.^9^ The method has been used extensively for the evaluation of health services and health technologies,^10^, ^11^ and there is increasing application in the determination of benefit–risk trade‐off for medicines.^12^, ^13^ In DCEs, respondents are asked to choose their preferred option from a set of hypothetical (but realistic) alternatives, on the basis of attributes and their respective levels determined using established qualitative methods.^14^

Modeling the expected utility of drugs, on the basis of patient‐defined benefit–risk trade‐offs, offers a quantitative approach to inform drug development and regulatory decision making. The present study demonstrates the potential for the DCE method in benefit–risk assessment. Patient priorities for drug outcomes are first identified using qualitative interviews and ranking exercises. Patient preferences for the most important outcomes are then valued using a DCE. This generates preference weights for each selected outcome that are applied to observed data from a clinical trial. The expected utilities of alternative drugs are then modeled to generate patient‐defined benefit–risk preferences for a selection of alternative drugs.

The case study is based on antiepileptic drugs (AEDs), selected because of the particular need to balance benefits against the risks of harm.^15^ Although two thirds of patients treated with AEDs will achieve remission from seizures within 5 years of diagnosis, at least 40% of patients will experience treatment‐related adverse effects.^16^, ^17^ Patient‐determined levels of acceptable harm for improved seizure control are rarely considered in clinical trials.^18^ However, they are essential considerations, especially for women of childbearing potential with generalized epilepsy, in whom choices need to be made between the most effective yet teratogenic treatment, valproate, and less teratogenic (but less effective) alternatives.^17^, ^19^ In this case study, we aimed to compare patient‐defined benefit–risk preferences with the results of a clinical trial of AEDs^16^, ^17^ and to assess differences between patient subgroups.

## Results

### Benefit and risk attribute selection

Fifty‐four patients and nine physicians (34% and 90% of those invited, respectively) participated in the qualitative study to identify the most important benefit and risk outcomes for use in the DCE. Reduction in seizure frequency was the most important treatment outcome (benefit) across all groups (**Table** **S1**), and discussions surrounding this outcome focused on seizures stopping and patients achieving remission. The importance of adverse effects (risks) varied by patient group: recently diagnosed patients were most concerned about feelings of aggression and depression, whereas those with an established diagnosis were most concerned about memory problems. Women of childbearing potential also ranked memory problems highest, followed by the risk of fetal abnormality if they became pregnant while taking the drug. Physicians ranked memory problems and depression highly. Patients and physicians valued life impacts (e.g., reduced independence), but in the context of the decision to initiate or switch an AED, physicians considered them to be consequences of epilepsy, or the adverse events (AEs) of treatment, rather than independent treatment outcomes. As such, they were excluded from the DCE. Attributes selected for the DCE are presented in **Table** 1. Two versions of the DCE were developed to represent the most important attributes selected by patients with a recent or established diagnosis (DCE‐1) and women of childbearing potential (DCE‐2) (**Figure** **S1**).

**Table 1:** Attributes and levels of the discrete choice experiment

DCE attribute^a^	DCE levels (coding)	Level selection	Description (before choice questions)
**Seizures stop** One year after starting this medication	5 in 10 people (0.5) 3 in 10 people (0.3)	Plausible estimates based on: Seizure frequency^16^, ^17^ Clinical expert opinion (research team, scientific advisory group, and group discussion meeting with prescribing physicians)	We would like you to imagine you have the choice between two medications: medication A and medication B. We will give you the same information about each medication. The chance of responding well: ‐ Seizures stop ‐ Fewer seizures
**Fewer seizures** One year after starting this medication	3 in 10 people (0.3) 1 in 10 people (0.1)		
**Memory problems** These problems frequently affect activities of daily life	1 in 100 people (0.01) 7 in 100 people (0.07)	Plausible estimates based on: Clinically important adverse events and patient‐reported quality of life outcomes^16^, ^17^ Section 4.8 of the summary of product characteristics of AEDs used in the SANAD trial^16^, ^17^ Clinical expert opinion (research team, scientific advisory group, and group discussion meeting with prescribing physicians)	The risk of severe adverse effects. ‐ Memory problems ‐ Depression ‐ Feelings of aggression^b^ These adverse effects^c^ would be so severe that you would need to change to a different antiepileptic medication
**Depression** A feeling of low mood that often affects activities of daily life	1 in 100 people (0.01) 8 in 100 people (0.08)		
**Feelings of aggression** b This can be verbal or physical and often affects relationships and activities of daily life	1 in 100 people (0.01) 8 in 100 people (0.08)		
**Harm to your fetus if you get pregnant while taking this medication** d Causing problems from birth, such as spina bifida or low IQ	2 in 100 pregnant women (0.02) 9 in 100 pregnant women (0.09)	Minimum and maximum risk of AED‐related fetal abnormality reported to patients via the Epilepsy Action charity website at the time of the survey.	Finally, we will also give you information on the risk of harm to the fetus if you get pregnant while taking this medication. This may cause problems, such as spina bifida, a hole in the heart, and a cleft palate (where the roof of the mouth is not correctly joined). This may also cause neurodevelopment problems, such as poor memory, poor language and social skills, and low IQ.

AED, antiepileptic drug; DCE, discrete choice experiment; IQ, intelligence quotient; SANAD, Standard vs. New Antiepileptic Drugs. ^a^As described in each of the eight choice questions. ^b^Only in DCE for patients with a recent or established diagnosis. ^c^Term “adverse effects” used to describe adverse events, as per findings of our qualitative study. ^d^Only in DCE for women of childbearing potential.

### Discrete choice experiment

Among those who consented to the survey, 4 withdrew before randomization and 29 did not start their DCE, leaving 280 patients for this analysis (**Table** 2).

**Table 2:** Patient characteristics

Characteristics	DCE‐1 (*N* = 177)		DCE‐2 (*N* = 103)	
	Excluding women of childbearing potential		Women of childbearing potential	
	*n*/*N*	% or range^a^	*n*/*N*	% or range^a^
Demographics				
Age, median, years^b^	45	18–79	29	18–55
Female sex	95/177	54	103/103	100
White British	140/149	94	84/86	98
Live alone	28/149	19	6/87	7
Employed	76/149	51	69/87	79
Time since diagnosis, years				
<1	9/176	5	3/103	3
1–10	40/176	23	40/103	39
>10	127/176	72	60/103	58
Seizure types				
Focal	56/157	36	40/96	42
Complex focal	70/157	45	45/96	47
Absences	64/157	41	46/96	48
Tonic clonic	102/157	65	73/96	76
Time since last seizure (<1 month)	88/159	56	48/95	51
Seizure frequency compared with 1 year ago				
Increased	39/157	25	17/96	18
Constant	69/157	44	48/96	50
Decreased	49/157	31	31/96	32
Change to antiepileptic medication (changes in past 3 months)	66/151	44	43/93	46
Change reason seizures	43/65	66	31/42	74
Change reason adverse effects	19/65	29	15/42	36
Change reason remission	5/65	8	2/42	5
Self‐reported nonadherence to antiepileptic medication^c^	37/66	56	24/42	57
Experience of adverse effects				
Aggression^c^	15/66	23	11/42	26
Depression^c^	15/66	23	16/42	38
Memory problems^c^	22/66	33	14/42	33
Change antiepileptic medication because of pregnancy concern	NA	NA	31/97	32

DCE‐1, discrete choice experiment for patients with a recent or established diagnosis; DCE‐2, DCE for women of childbearing potential; NA, not applicable. ^a^Percentage of eligible responses (excluding missing data). ^b^ *N* = 177 (DCE‐1), *N* = 103 (DCE‐2). ^c^Number restricted to patients who reported a change in the type or amount of antiepileptic drug in the past 3 months, because of a routing error in the online survey.

All patients preferred the AED offering the greatest benefits and the lowest risk of harm, as indicated by direction of the signs on the coefficients (+/−) (**Table** 3). Patients had stronger preferences for reductions in the risk of AEs than improvements in 12‐month seizure remission, as shown by the greater magnitude of the harm coefficients. Experiencing fewer seizures did not have a significant influence on AED preferences of women of childbearing potential (*P* = 0.685).

**Table 3:** Results of the DCE random‐effects logistic regression model

Attribute	DCE‐1: excluding women of childbearing potential^a^			DCE‐2: women of childbearing potential^a^		
	Coefficient (95% CI)^b^	*P* value	Maximum acceptable incremental risk (%) per 1% increase in 12‐month remission (95% CI)^b^	Coefficient (95% CI)^b^	*P* value	Maximum acceptable incremental risk (%) per 1% increase in 12‐month remission (95% CI)^b^
Remission	0.03 (0.03 to 0.05)	<0.001	NA	0.05 (0.04 to 0.07)	<0.001	NA
Fewer seizures	0.01 (0.00 to 0.02)	0.010	NA	−0.00 (−0.01 to 0.01)	0.685	NA
Depression	−0.11 (−0.15 to −0.10)	<0.001	0.31 (0.24 to 0.39)	−0.08 (−0.13 to −0.06)	<0.001	0.56 (0.38 to 0.88)
Memory problems	−0.11 (−0.16 to −0.10)	<0.001	0.30 (0.23 to 0.40)	−0.14 (−0.21 to −0.11)	<0.001	0.34 (0.26 to 0.45)
Aggression/fetal abnormality	−0.13 (−0.18 to −0.13)	<0.001	0.25 (0.20 to 0.31)	−0.23 (−0.32 to −0.21)	<0.001	0.20 (0.16 to 0.24)
Constant	0.03 (−0.19 to 0.12)	0.689	NA	0.47 (0.25 to 0.92)	<0.001	NA
Number of observations	1,339			790		
Number of groups	177			103		
Wald χ^2^ (5 degrees of freedom)	321.27			154.55		
Log likelihood	−674.71			−360.77		
Pseudo *R* ^2^	0.27			0.34		
*P* value	<0.000			<0.000		

CI, confidence interval; DCE, discrete choice experiment; DCE‐1, DCE for patients with a recent or established diagnosis; DCE‐2, DCE for women of childbearing potential; NA, not applicable. ^a^A total of 159 respondents to DCE‐1 (90%) and 97 respondents to DCE‐2 (94%) passed the dominance check question included for internal validity, by selecting the antiepileptic drug aligned with *a priori* expectations and thus indicating they comprehended the task. A model excluding respondents who failed the dominance check for internal validity did not differ significantly. ^b^CIs generated by 1000 bootstrap replications.

The maximum acceptable increase in risks of adverse effects for an AED that increases the chance of 12‐month remission by 10% (in DCE‐1) were as follows: 3.1% for depression, 3.0% for memory problems, and 2.5% for aggression. For women of childbearing potential, the MAR of fetal abnormality was 2.0% for a 10% increase in probability of remission, compared with 5.6% for depression and 3.4% for memory problems.

### Patient utility associated with selected AEDs

**Figure 1:**
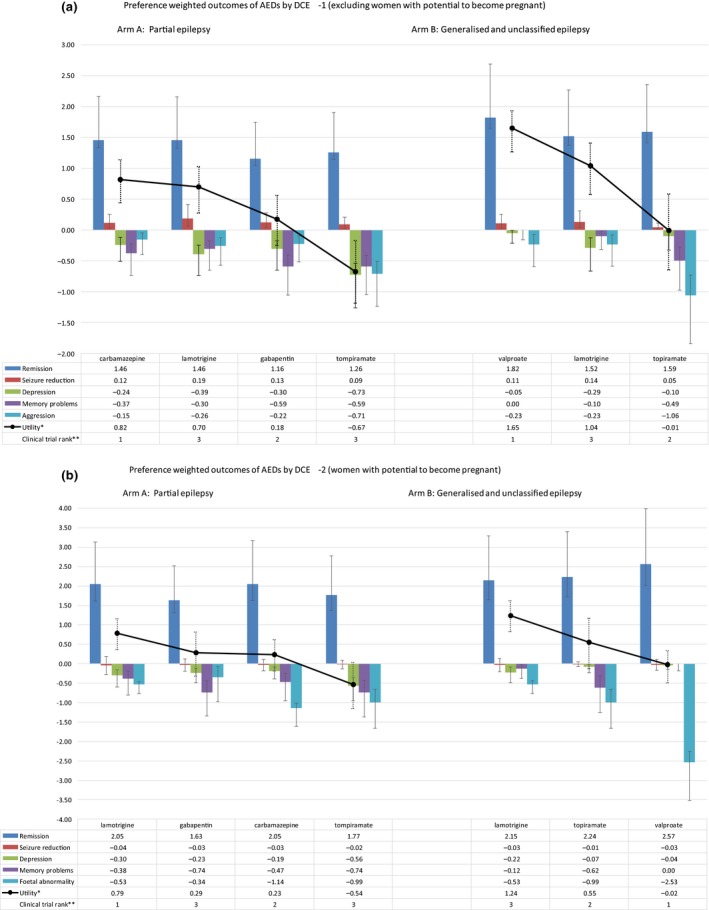
AED, antiepileptic drug; DCE‐1, discrete choice experiment for patients with a recent or established diagnosis; DCE‐2, DCE for women of childbearing potential. Preference‐weighted outcomes of AEDs by DCE‐1 (**a**) and DCE‐2 (**b**). *High utility is most preferred. AEDs presented in order of highest to lowest utility, left to right by indication. **Clinical rank based on primary outcome in Standard vs. New Antiepileptic Drugs (SANAD) I (time to treatment failure). Rank 1 = high (i.e., longest time to treatment failure).

The preference‐weighted outcomes (remission, reduction, memory problems, depression, aggression/fetal abnormality) for four alternative drugs for focal epilepsy (carbamazepine, lamotrigine, gabapentin, and topiramate) and three alternative drugs for generalized and unclassified epilepsy (valproate, lamotrigine, and topiramate)^16^, ^17^ are displayed in **Figure** 1. On the basis of total utility (sum of the weighted outcomes), respondents to DCE‐1 indicated carbamazepine would be the most preferred AED for focal epilepsy, and topiramate the least. The disutility associated with topiramate (−0.67) suggests these patients would prefer to avoid it. For generalized or unclassified epilepsy, valproate yielded higher utility than both lamotrigine and topiramate. In both cases, ranking based on utility differed from ranking based on time to treatment failure, as observed in clinical trials (**Figure** 1 **a**); however, this did not reach statistical significance.

Women of childbearing potential responding to DCE‐2 favored lamotrigine over carbamazepine for focal epilepsy, in agreement with trial‐based ranking; however, gabapentin ranked second when the trial outcomes were weighted in the utility model. Topiramate was associated with the lowest utility. Lamotrigine was also favored for generalized or unclassified epilepsy, with valproate being the least preferred; thus, weighting by patient preferences for benefit and harm outcomes resulted in a statistically significant reversal of rankings.^20^ The combined risk and preference weighting for fetal abnormality heavily influenced the disutility of valproate (**Figure** 1 **b**).

The improvement in benefit associated with a switch to valproate did not outweigh the increase in risk for women of childbearing potential. A switch from topiramate to valproate for generalized or unclassified epilepsy, for instance, increased the risk of fetal abnormality by 6.65%,^19^ which exceeds the 1.41% MAR that corresponds to the 7% improvement in 12‐month remission seen with valproate^17^ (**Table** **S2**).

## Discussion

DCEs represent a valid and reliable method to quantify patient‐focused outcomes.^10^, ^11^, ^12^, ^13^ The utility model demonstrated how quantitative data on patient preferences can be integrated with clinical evidence to provide a patient‐focused benefit–risk analysis that could be used to inform regulatory decision making. The case study illustrates how DCEs can provide an explicit estimate of patient‐defined MAR, which may differ from value judgements based on clinical evidence alone.

**Figure 2:**
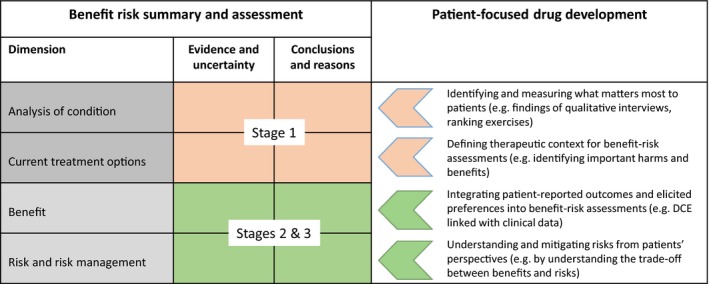
DCE, discrete choice experiment; FDA, US Food and Drug Administration. Adaptation of the FDA Benefit–Risk Framework, highlighting opportunities for consideration of patient‐focused evidence across each dimension, and linking to methods described for stages 1, 2, and 3 in the context of antiepileptic drug assessment.

The FDA implemented changes after the fifth authorization of the Prescription Drug User Fee Act, to increase the clarity, transparency, and consistency of its benefit–risk assessments. With the passage of the 21st Century Cures Act, the FDA now has an imperative to consider how relevant patient experience data and related information can be incorporated into the structured benefit–risk assessment framework to inform regulatory decision making.^6^ Our proposed method is one approach that might address the FDA's recognized need to use more systematic, methodologically sound approaches to collect patient input so that it becomes data that can further inform regulatory decision making^21^ (**Figure** 2).

Patients have an important role alongside all other stakeholders in determining relevant outcomes and priorities, acceptable uncertainty, as well as benefit–risk and value of a medicine.^1^, ^2^ However, patients believe that decision makers, particularly regulators, do not always have a complete understanding of the risks that patients with some illnesses are willing to accept, and that this benefit–risk assessment will vary by disease.^22^ Our DCE‐based approach linked with clinical trial evidence addresses this, by generating patient‐defined benefit–risk thresholds for drugs for specific clinical indications.

There are several examples of what could be interpreted as different levels of acceptance of risk among some patients, regulators, or other stakeholders–most notably, in the context of HIV, where “patient experts” successfully challenged the drug development and licensing paradigms, leading up to the FDA's Accelerated Approval pathway. The antiparkinsonian drug, tolcapone, was marketed in the European Union in 1997, but cases of fatal hepatotoxicity led to its marketing authorization being suspended the following year.^23^ Patients and physicians argued that some patients experience improved quality of life and pleaded to gain access to the drug while explicitly accepting the risk of hepatotoxicity. Their lobbying led to the lifting of the suspension in 2004. Both natalizumab (for patients with relapsing forms of multiple sclerosis) and alosetron (for irritable bowel syndrome) were voluntarily withdrawn by their manufacturers because of safety concerns.^23^ However, the FDA recommended their reintroduction to the market at the request of patients and family members. The use of methods to support regulatory benefit‐risk assessments that consider patient preferences explicitly might have lessened the likelihood of misaligned preferences between the various stakeholders. Benefit–risk assessments are a qualitative exercise grounded in quantitative evidence, but just because patient perspectives are subjective does not mean that they cannot be quantified and analyzed accordingly.

Our case study showed that although seizure freedom was ranked the most important treatment outcome by patients during qualitative interviews, patients also prioritized reduction in the risk of adverse effects, and the importance of these effects differed by patient subgroup. The results of the DCE, however, suggest that patients are willing to accept a reduction in the chance of remission in exchange for a reduction in the risk of adverse effects—and that they attach a higher value on improving risk reduction than benefit. Women of childbearing potential would accept 5% reduction in the probability of 12‐month remission to reduce the risk of fetal abnormality by 1%. The potential for our method to identify both MAR, and willingness to forego benefit, is particularly important with respect to prescribing valproate, which is considered the most effective treatment for certain epilepsy syndromes, such as juvenile myoclonic epilepsy, but is associated with the highest risk of teratogenicity.^24^

This study provides quantitative evidence on women's strength of preference to avoid valproate, which aligns with recent advice from regulatory authorities.^25^, ^26^, ^27^ Understanding the balance between benefit and harm, and the extent to which women are willing to forego benefit for this reduction in harm, could assist in the development of recommendations of alternative treatments to valproate and inform parameters of equivalence for new drugs.

We are aware of previous use of DCE to elicit preferences for AEDs.^28^, ^29^, ^30^, ^31^ Lloyd *et al*.^28^ found that UK patients were willing to forego 4.85% seizure control for a 1% reduction in risk of hair loss, 4.45% seizure control for a 1% reduction in risk of skin rash, and 1.37% seizure control for a 1‐lb (0.45‐kg) reduction in weight gain. Manjunath *et al*.^29^ measured US patient preferences for add‐on AEDs in terms of seizure frequency and AEs; and they found that seizure reduction was the top priority when ranked against the reduction or elimination of AEs. Powell *et al*.^30^ compared patients’ and neurologists’ preferences for a carbamazepine pharmacogenetic testing service. Ettinger *et al*.^31^ also conducted a DCE in patients and neurologists, and they found that, although both ranked seizure control most important, neurologists’ preferences were more influenced by seizure reduction compared with adverse effects for patients. None of these studies, however, assessed preferences in the context of trial data pertaining to actual AEDs; in fact, we are unaware of any other studies that integrate benefit–risk preference weights, estimated using DCE methods, and trial data.

The key strengths of our study are in meeting the recommended qualities of patient preference studies specified by the CDRH^5^: (i) *Patient centeredness*: the DCE measured patient preferences for outcomes. Furthermore, the study encompassed the views of both patients and physicians at appropriate stages of developing the DCE, to ensure the hypothetical task was relevant to the context of clinical decisions. (ii) *Representativeness of the sample and generalizability of results*: the study used an appropriate sample size in each clinically well‐defined group; however, there may be limitations in generalizability because of the sampling. (iii) *Capturing heterogeneity of patients’ preferences*: we identified clinically important subgroups *a priori* and tailored individual DCEs in response to heterogeneity in preferences at the qualitative phase. (iv) *Established good research practices by recognized professional organizations*: we adhered to good practice guidelines.^14^, ^32^ (v) *Effective communication of benefit, harm, risk, and uncertainty*: attribute levels were displayed as natural frequencies with pictographs, as recommended.^5^ (vi) *Minimal cognitive bias*: cognitive interviewing was used to minimize framing effects and choices ordered randomly. (vii) *Logical soundness*: we demonstrated a high level of internal validity with a dominance test. (viii) *Relevance*: potential attributes identified from validated outcome measures, reduced systematically using a qualitative study and defined by clinically meaningful thresholds. (ix) *Robustness of analysis of results*: analysis adhered to good practice guidelines^9^, ^32^ with uncertainty represented by bootstrapped confidence intervals. (x) *Study conduct*: trained and experienced researchers interviewed patients, and the DCE questionnaire was verified by patients and healthcare professionals. (xi) *Comprehension by study participants*: robust application of cognitive interviews and questionnaire piloting to ensure face validity and optimal comprehension choice tasks.^14^

There were, however, limitations to our approach. Bias arising from patients self‐selecting to complete the questionnaire will limit the generalizability of the results. We also acknowledge that although DCE is a recommended approach to benefit–risk assessment, other methods exist, including multicriteria decision analysis^33^ and health outcome modeling.^8^, ^34^ Our methods highlighted differences in responses linked to the methods used. The ranking of single outcomes identified seizure remission as the most important, whereas the DCE identified that reductions in the risk of AEs were more highly valued than improvements in seizure control. Although ranking exercises do not consider trade‐offs explicitly, DCEs benefit from capturing this to assess the MAR for a given level of benefit. DCEs are inherently limited by the potential for patients’ stated preferences not reflecting their revealed preferences accurately and the cognitive burden placed on respondents, which increases rapidly with the number of attributes. Consequently, differences between studies in benefit and risk attributes are inevitable,^28^, ^29^, ^30^, ^31^ although in mitigation, our study used a robust approach to attribute selection.^5^, ^14^, ^32^

In conclusion, our case study findings indicate that accounting for patient preferences, in addition to clinical variables, could lead to different treatment choices or regulatory decisions. The most substantive results for AEDs are that women of childbearing potential show a clear preference for lamotrigine over valproate, principally driven by a preference to avoid the risk of teratogenicity (major malformation rate, ≈11%^19^). This is despite clinical trial results showing valproate to be significantly more effective than lamotrigine in generalized onset seizures,^17^ and aligns with recommendations from regulators.^25^, ^26^

The study demonstrates how patients’ preference for treatment benefits and risks can be incorporated into benefit–risk assessments by obtaining quantitative evidence using a DCE. Our method represents a transparent and valid approach to examining a patient‐oriented perspective in support of regulatory assessments of medicines.^1^ It also builds on previous research that highlighted that, although studies quantifying preferences may allow for formal evidence‐based appraisal of benefit–risk values, more is required to ensure best practice and to develop methods that combined stated preferences and clinical data.^35^ Certainly, drug regulators will need to be cognizant of the evolving methods of preference‐based benefit–risk, including DCEs.^36^

## Methods

The study comprised three stages (**Table** 4): (i) identification of the most important outcomes of AED treatment using qualitative interviews and ranking exercises with patients and a focus discussion group meeting of physicians; (ii) elicitation of patient preferences for these outcomes using a DCE; and (iii) estimation of the expected utility associated with alternative AEDs by combining preference weights with data from a clinical trial.

**Table 4:** Summary of the stages involved in development of the case study

Stage	Stage 1: benefit–risk outcome selection		Stage 2: DCE	Stage 3: modeling‐expected utility of alternative AEDs	
Principal (supplementary) method	Interviews with patients	Meeting with physicians (cognitive interviews with patients)	Web‐based survey (pilot questionnaire)	Obtain observed data for outcomes	Calculate expected utility model
Aim	To identify the outcomes of AEDs that are most important to patients and their priorities for these outcomes	To assess the plausibility of the benefit–risk outcomes selected by patients in stage 1, for use in a DCE (to confirm the face validity of the highest rank outcomes in the format of a DCE)	To value the patient preferences for five benefit–risk outcomes of AEDs	Obtain trial data on outcome event rates for four alternative drugs for focal epilepsy (carbamazepine, lamotrigine, gabapentin, and topiramate) and three alternative drugs for generalized and unclassified epilepsy (valproate, lamotrigine, and topiramate)	Estimate the expected utility associated with each AED for both indications
Sample	Adult patients with epilepsy recruited via three specialist neurology secondary and tertiary care referral centers in England (*n* = 41). Subgroups: patients with a recent diagnosis (3–12 months), patients with an established diagnosis (>12 months), and women of childbearing age (18–50 years)	Nine physicians responsible for prescribing AEDs to adults with epilepsy (a further 13 patients from sampling frame used in stage 1)	Adult patients self‐reporting as aged ≥18 years and diagnosed with epilepsy by a physician (*n* = 280). Subgroups: women self‐identifying as being of childbearing potential and other responders (Epilepsy Action staff and volunteers, clinical and academic researchers, and members of the scientific advisory group)	SANAD clinical trial and Cochrane review of monotherapy treatment of epilepsy in pregnancy	
Method	Semistructured interview and ranking exercise: Which outcomes are most important to you? Rank your top four (highest = 4, lowest = 1). Analysis: Mean rank score per outcome by subgroup. Outputs: Most important outcomes from patients’ perspective	Group discussion and individual ranking exercises: Which outcomes are most important to you? Define the frequency and seriousness at which an adverse event becomes “clinically important.” Analysis: Mean rank score per outcome by time since patient diagnosis, early or established. Outputs: Most plausible outcomes from the prescribers’ perspective (think aloud experiment)	Random‐effects logit model with 1,000 bootstrap replications. Outputs: preference weights for outcomes, maximum acceptable risk of harm for a gain in benefit (Online: invitation to complete the questionnaire and supply comments)	Parameter uncertainty represented by drawing from β distributions for the number of events in the observed data. 1,000 replications were simulated, and the confidence intervals were taken to be the 25th and 975th percentile of the variable	Utility = Σ(β_outcome*events). Confidence intervals generated from simulated preference and event data. Outputs: Total utility and rank order of AEDs by preference‐weighted rank
Outcomes assessed at each stage	*Benefits:* reduction in seizure frequency, reduction in seizure severity*Adverse events:* memory problems, depression, fetal abnormality, anger and aggression, headache, sleepiness and drowsiness, difficulty concentrating, weight gain, skin rash, dizziness, nervousness and/or agitation, tiredness *Life impacts:* limits ability to work in paid employment, reduces independence, negative impacts on relationships with family and/or friends, makes you feel less in control of the things that happen to you, limits hopes and plans for the future, limits social life and activities, increases the worry about having a seizure, causes problems with everyday memory and/or concentration, extent to which other people treat you like an inferior person, makes you feel more negative about yourself Patients could also self‐nominate any outcome they considered to be missing	*Benefits:* reduction in seizure frequency *Adverse events:* memory problems, depression, fetal abnormality, anger and aggression, headache *Life impacts:* limits ability to work in paid employment, reduces independence, negative impacts on relationships with family and/or friends, makes you feel less in control of the things that happen to you, limits hopes and plans for the future	*Benefits:* remission, reduction in seizure frequency *Adverse events:* memory problems, depression, anger and aggression, fetal abnormality	*Benefits:* remission, reduction in seizure frequency *Adverse events:* memory problems, depression, anger and aggression, fetal abnormality	*Benefits:* remission, reduction in seizure frequency *Adverse events:* memory problems, depression, anger and aggression, fetal abnormality Total utility per AED

AED, antiepileptic drug; DCE, discrete choice experiment; SANAD, Standard vs. New Antiepileptic Drugs.

Ethical approval was granted by the UK National Health Service Research (reference number: 11/NW/0191).

### Stage 1: benefit–risk outcome selection

The first stage in the method was to identify patients’ most important outcomes of AED treatment for inclusion in the DCE. A qualitative study was designed according to good practice guidelines.^14^, ^32^, ^37^ All interviews were conducted in patients’ own homes, and consent was requested to audiotape record for subsequent transcription. Patients were asked to consider predefined treatment outcomes, which were categorized according to whether they were treatment benefits, adverse effects, or life impacts, and selected from clinical trials^16^, ^17^ and validated outcome measures.^38^, ^39^, ^40^, ^41^ Patients were first asked to nominate any additional outcomes, on the basis of their own experiences, that they considered were missing from each category. Next, they were asked to select the outcomes that were most important to them from each category. Finally, considering all the outcomes they had selected, across the three categories, they were asked to choose their top four, and rank them in order of importance.

The face validity and plausibility of the 10 most important attributes overall were examined using cognitive interviews with patients and a meeting with prescribing physicians. The meeting of physicians responsible for prescribing AEDs to adults with epilepsy was convened at the Walton Centre National Health Service Foundation Trust. Participants were first asked to rank the 10 treatment outcomes that patients had considered the most important. They then participated in semistructured discussions to share their practical experience of discussing treatment outcomes with patients and, in particular, their distinction between “adverse effects” and “life impacts” relating to AEDs. They were asked to independently record the frequency and seriousness at which an AE becomes a “clinically important” AE that required a change in treatment. For example, clinically important depression was described as “low mood which often affects activities of daily life.” The purpose of this exercise was to ensure parity between DCE descriptions of treatment outcomes and clinically important AE data recorded in clinical trials,^16^, ^17^ to ensure optimal integration of preferences with clinical evidence. Prescribers were also asked to draw on their experience of communicating benefit–risk to patients and to provide feedback on the formatting of the patient DCE questionnaire. Discussions were audiotape recorded, and ranking results were noted in workbooks that were self‐completed during the session.

### Stage 2: DCE

The DCE was conducted, analyzed, and reported according to good practice guidelines.^32^, ^42^ The five treatment outcomes ranked highest by patients, and which were considered clinically plausible, were included as attributes in the DCE. Each attribute was assigned two levels on the basis of data from a clinical trial^16^, ^17^ and information on the risk of AED‐related fetal abnormality available to patients on the Epilepsy Action charity website at the time of the survey. Levels represent the frequency of events observed in clinical trials and were presented in the questionnaire as a “1 in *X* people experience…”, supplemented with pictograms that used a traffic light color coding for positive and negative effects.

The DCE consisted of eight binary choice scenarios (**Figure** **S1**), which asked: Which medication would you prefer? A partially dominant choice was used to test the internal validity of the DCE, in which “medication A” had a higher chance of remission and lower risk of AEs; therefore, it was assumed respondents would prefer this option.

#### Patient DCE questionnaire

Adults self‐reporting as being aged ≥18 years and diagnosed as having epilepsy by a physician were eligible to complete the survey. Respondents were required to consent to participate in the study before they accessed the questionnaire, and there was no compensation for their time. Recruitment was via publicity from the charity, Epilepsy Action, an advertisement in local press, and posters in 113 National Health Service outpatient clinics across England and Wales. The survey was implemented online (Snap Surveys, London, UK) between June and October 2013 and hosted by the Epilepsy Action website. Estimated completion time was 30 minutes. Target sample size was a minimum of 63 respondents per DCE, based on each main effect level of interest being represented across the design at least 500 times.^43^ A random sample of 25% was directed to a DCE designed to compare patients’ with physicians’ preferences for pharmacogenetic testing prior treatment with carbamazepine, which is reported elsewhere.^30^ Remaining respondents were directed to one of the two DCEs reported herein via a series of filter questions (DCE‐2 for women of childbearing potential; DCE‐1 for everyone else).

The questionnaire was piloted in a convenience sample of Epilepsy Action staff and volunteers, clinical and academic research staff, physicians who agreed to be contacted after the focus group, and members of our scientific advisory group. These individuals provided feedback on aspects such as the phrasing of the attributes and the selection criteria for women of childbearing potential, which was changed from being defined by age to a single question that asked, “Is there any chance, however remote, that you may become pregnant in the future?”

#### Statistical analysis

Responses to the DCE were analyzed in STATA, version 13 (StataCorp LP, College Station, TX) using a random‐effects logit model that allowed for multiple observations (eight binary choices) from the same respondent.^44^ The regression model estimated preference weights for each attribute that indicate the importance of attributes and the direction of effect. The coefficients (β) from the regression were used to calculate the MAR of an AE that respondents were willing to accept in exchange for a percentage point improvement in benefit (12‐month remission) (**Figure** **S2**).

Confidence intervals (95%) were determined from 1000 bootstrap replications.

### Stage 3: estimating the utility for a given AED

The expected utility model was parameterized using the preference weights elicited in the DCE and data on the number of events for each outcome assessed in the DCE, from the Standard vs. New Antiepileptic Drugs (SANAD) clinical trial of AEDs^16^, ^17^ and from a meta‐analysis of the effects of prenatal exposure to commonly prescribed AEDs on the prevalence of congenital malformations in the child^19^ (**Table** 5). The β coefficients for each outcome (derived in stage 2) were multiplied by the corresponding clinical trial event rate and summed to estimate the total utility for each AED^42^ (**Figure** **S2**). The ranking of AEDs by total utility was compared with ranking by time to treatment failure (because of inadequate seizure control or AEs), chosen to reflect the benefit–harm trade‐off observed in the clinical trial.^16^, ^17^

**Table 5:** Clinical event data used to calculate preference weights and total utility by AED

Variable	Partial epilepsy					Generalized and unclassified epilepsy			
	Carbamazepine	Gabapentin	Lamotrigine	Topiramate	Ref.	Valproate	Topiramate	Lamotrigine	Ref.
12‐Month remission at year 2 observation (PP)	44	35	44	38	16	55	48	46	17
Seizure reduction at year 2 observation^a^	13	14	21	10	16	12	5	15	17
Clinically important adverse effects									
Depression	2.23	2.79	3.58	6.70	16	0.44	0.88	2.64	17
Memory problems	3.35	5.31	2.75	5.31	16	0.00	4.42	0.88	17
Behavior/personality change/aggression	1.12	1.68	1.93	5.31	16	1.75	7.96	1.76	17
Major congenital malformation outcomes in the children of women receiving AED treatment while pregnant	4.93	1.47	2.31	4.28	19	10.93	4.28	2.31	19

Data are given as events per 100 patients.

AED, antiepileptic drug; PP, trial per‐protocol analysis.

a Calculated as the number of patients still receiving randomized drug at year 2 observation, minus the number of patients who had achieved 12‐month remission at year 2 observation (based on PP).

## Supporting information

Supplementary information accompanies this paper on the *Clinical Pharmacology & Therapeutics* website (www.cpt-journal.com).

## Funding

Funded by the National Institute for Health Research (NIHR), under its Research for Patient Benefit Programme (Grant Reference Number PB‐PG‐0909‐20161). P.W., A.M., and D.A.H. are also supported by the Medical Research Council North West Hub for Trials Methodology Research (Reference Number MR/K025635/1). A.M. is partly funded by the NIHR Collaboration for Leadership in Applied Health Research and Care North West Coast; and D.A.H. is a Health and Care Research Wales Senior Research Leader (SRL/15/029).

## Conflict of Interest

The authors declared no competing interests for this work. The funder had no role in study design; in the collection, analysis, and interpretation of data; in the writing of the report; or in the decision to submit the paper for publication.

## Author Contributions

E.H. and D.H. wrote the manuscript; A.M., D.H., E.H., A.J., P.W., and G.B. designed the research; E.H., A.R., D.H., and A.M. performed the research; E.H. and C.P. analyzed the data.

## Supporting information


**Table S1** Results of the ranking exercises, presented as standardized weighed rank scoresa.**Table S2** Maximum acceptable risk (MAR) and difference in total utility associated with a change to AED with better seizure control.**Figure S1** Example binary DCE question.**Figure S2** Model specification and analysis.
