# Supplementary material for: Patient‐Focused Drug Development Methods for Benefit–Risk Assessments: A Case Study Using a Discrete Choice Experiment for Antiepileptic Drugs
Source: Clin Pharmacol Ther. 2018 Oct 25;105(3):672–83. doi: 10.1002/cpt.1231 (PMC6491963; doi:10.1002/cpt.1231)
Supplement: Supplementary file 1 — Table S1 Results of the ranking exercises, presented as standardized weighed rank scoresa.Table S2 Maximum acceptable risk (MAR) and difference in total utility associated with a change to AED with better seizure control.Figure S1 Example binary DCE question.Figure S2 Model specification and analysis. [file CPT-105-672-s001.pdf]

Table S1: Results of the ranking exercises, presented as standardised weighed rank scores[a]

		Patient sub-group[b]			Physicians treating patients with:	
**Outcome**	**Outcome type[c] (reference)**	**Women of Childbearing Potential**	**Patient Recent Diagnosis**	**Patient Established Diagnosis**	**Recent Diagnosis**	**Established Diagnosis**
		n=18	n=10	n=13	n=9	
Reduction in seizure frequency[d]	B (16,17)	2.50	2.40	2.23	3.75	3.63
Memory Problems	AE (37,38)	1.22	0.10	0.69	1.56	1.63
Depression	AE (37,38)	0.22	1.10	0.08	1.35	1.60
Foetal Abnormality	AE (19)	0.50	n/a	n/a	excluded[e]	excluded[e]
Anger & aggression	AE (40)	0.22	1.60	0.00	1.06	1.13
Limits ability to work in paid employment	LI (39)	0.44	0.90	1.15	0.73	0.73
Reduces independence	LI (39)	0.78	0.80	0.31	0.54	0.54
Negative impacts on relationships with family and/or friends	LI (39)	0.28	0.30	1.00	0.81	0.56
Reduction in seizure severity	B (40)	1.17	0.30	0.85	_	_
Makes you feel less in control of the things that happen to you	LI (37)	0.56	0.30	0.62	0.00	0.00
Limits hopes & plans for the future	LI (39)	0.44	0.50	0.54	0.00	0.00
Limits social life and activities	LI (39)	0.11	0.50	0.69	_	_

Increases the worry about having a seizure	LI (37)	0.33	0.60	0.23	–	–
Headache	AE (38)	0.00	0.10	0.38	0.19	0.19
Causes problems with everyday memory and/or concentration	LI (37,38)	0.44	0.10	0.00	–	–
Sleepiness & drowsiness	AE (38)	0.06	0.40	0.00	–	–
Extent to which other people treat you like an inferior person	LI (37,39)	0.22	0.00	0.23	–	–
Difficulty concentrating	AE (37,38)	0.06	0.00	0.23	–	–
Weight gain	AE (38)	0.22	0.00	0.00	–	–
Skin rash	AE (38)	0.17	0.00	0.00	–	–
Dizziness	AE (38)	0.00	0.00	0.15	–	–
Makes you feel more negative about yourself	LI (39)	0.11	0.00	0.00	–	–
Nervousness and/or agitation	AE (38)	0.06	0.00	0.00	–	–
Tiredness	AE (38)	0.06	0.00	0.00	–	–

[a] Standardised score = Σ_rankscores / n. Rank scoring: highest rank = 4 points, lowest rank = 1 point. Maximum possible score=4.
[b] A further 13 patients completed the cognitive interviews to test the face validity of the DCE
[c] B = Benefit; AE = Adverse Event; LI = Life Impact; (#) = citation number in main manuscript
[d] Phrased as “seizure reduction” in the focus group with physicians
[e] Foetal abnormality is a mandatory consideration in AED prescribing practice
– Outcome did not qualify for ranking by physicians, i.e. not in top ten most important outcomes by patients

Table S2: Maximum acceptable risk (MAR) and difference in total utility associated with a change to AED with better seizure control

	Difference in % of 12-month remission compared to AED with better seizure control[16,17]	Maximum acceptable increase in AE risk associated with change to AED with better seizure control			Utility difference compared to AED with better seizure control[a]
		Depression	Memory Problems	Aggression / Foetal Abnormality	
**DCE 1: excluding women of childbearing potential**					
Monotherapy for focal epilepsy: Lamotrigine 12-month remission at Year 2 = 44%					
topiramate to lamotrigine	6	1·84	1·80	1·49	1·37
gabapentin to lamotrigine	9	2·76	2·70	2·24	0·53
carbamazepine to lamotrigine	0	0·00[b,c]	0·00	0·00[b,c]	-0·11
Monotherapy for generalised and unclassified epilepsy: Valproate 12-month remission at Year 2 = 55%					
topiramate to valproate	7	2·14	2·10	1·74	1·66
lamotrigine to valproate	9	2·76	2·70	2·24	0·61
**DCE 2: women of childbearing potential**					
Monotherapy for focal epilepsy: Lamotrigine 12-month remission at Year 2 = 44%					
topiramate to lamotrigine	6	3·33	2·01	1·21	1·33
carbamazepine to lamotrigine	0	0·00[b,c]	0·00	0·00[b,c]	0·56
gabapentin to lamotrigine	9	5·00	3·02	1·82	0·50
Monotherapy for generalised and unclassified epilepsy: Valproate 12-month remission at Year 2 = 55%					
topiramate to valproate	7	3·89	2·35	1·41[c]	-0·57
lamotrigine to valproate	9	5·00	3·02	1·82[c]	-1·26

[a] Negative utility difference indicates that patient would prefer to remain on existing therapy.

[b] 12-month remission equal for carbamazepine to lamotrigine therefore, change rejected if AE >with lamotrigine.

[c] Prevalence of AE with new AED (lamotrigine or valproate) exceeds MAR threshold, therefore change would be reject based on the benefit-risk ratio between 12-month remission and this AE.

Figure S1: Example binary DCE question

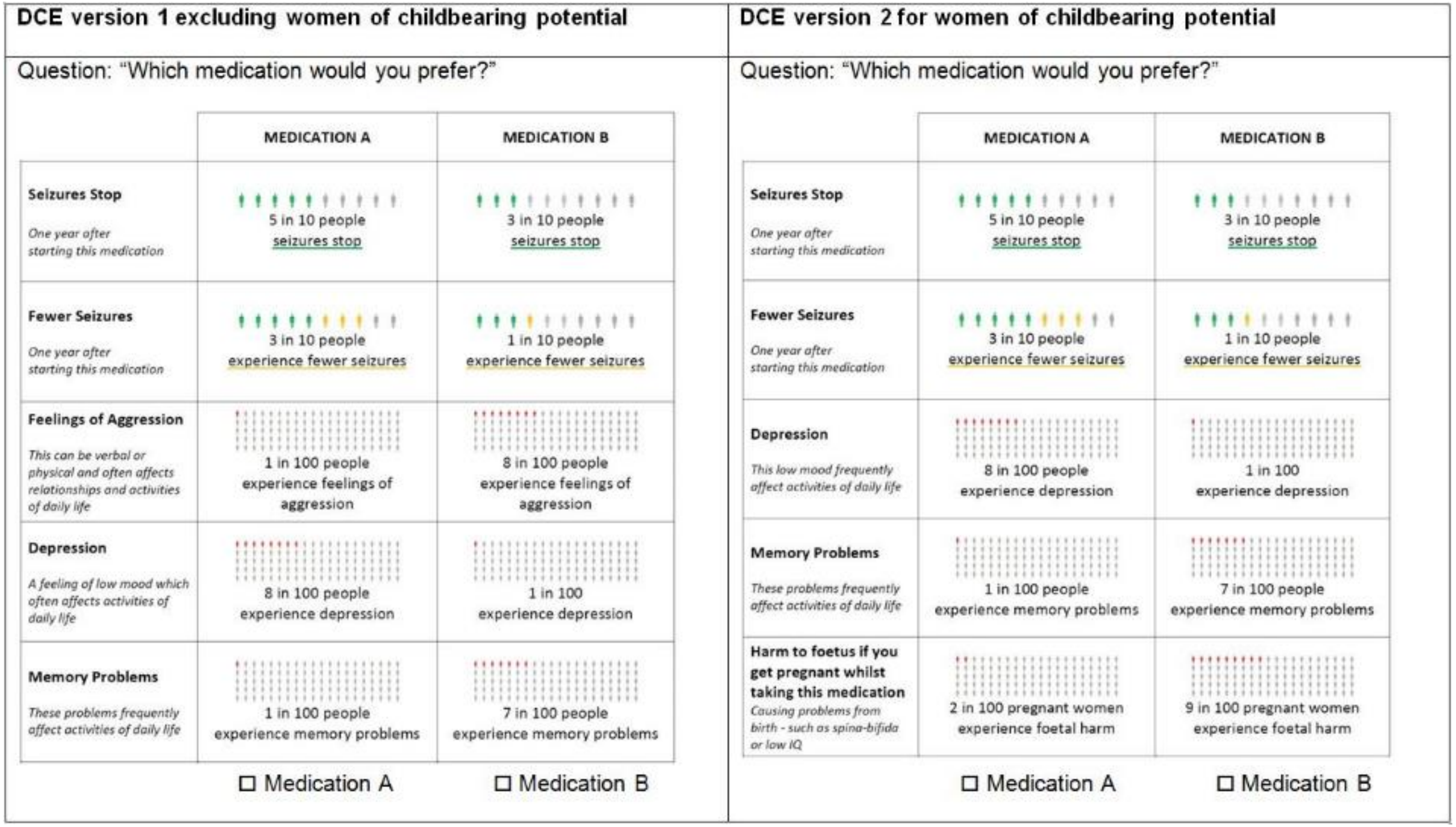

DCE version 1 excluding women of childbearing potential

Question: "Which medication would you prefer?"

	MEDICATION A	MEDICATION B
**Seizures Stop** *One year after starting this medication*	5 in 10 people seizures stop	3 in 10 people seizures stop
**Fewer Seizures** *One year after starting this medication*	3 in 10 people experience fewer seizures	1 in 10 people experience fewer seizures
**Feelings of Aggression** *This can be verbal or physical and often affects relationships and activities of daily life*	1 in 100 people experience feelings of aggression	8 in 100 people experience feelings of aggression
**Depression** *A feeling of low mood which often affects activities of daily life*	8 in 100 people experience depression	1 in 100 experience depression
**Memory Problems** *These problems frequently affect activities of daily life*	1 in 100 people experience memory problems	7 in 100 people experience memory problems

☐ Medication A ☐ Medication B

DCE version 2 for women of childbearing potential

Question: "Which medication would you prefer?"

	MEDICATION A	MEDICATION B
**Seizures Stop** *One year after starting this medication*	5 in 10 people seizures stop	3 in 10 people seizures stop
**Fewer Seizures** *One year after starting this medication*	3 in 10 people experience fewer seizures	1 in 10 people experience fewer seizures
**Depression** *This low mood frequently affect activities of daily life*	8 in 100 people experience depression	1 in 100 experience depression
**Memory Problems** *These problems frequently affect activities of daily life*	1 in 100 people experience memory problems	7 in 100 people experience memory problems
**Harm to foetus if you get pregnant whilst taking this medication** *Causing problems from birth - such as spina-bifida or low IQ*	2 in 100 pregnant women experience foetal harm	9 in 100 pregnant women experience foetal harm

☐ Medication A ☐ Medication B

A full factorial design (that included all possible combinations) would yield 32 AED profiles; therefore in order to keep the task manageable a fractional factorial design (plan 4a)[i] that contained eight profiles to define "Medication A", was used. Eight binary choices were generated by selecting the alternative level for "Medication B"[ii].

Figure S2: Model specification and analysis

Utility model[a]

Random effects logistic regression was used to estimate the parameters of the following utility model[43]:

$$U_{AED\_DCE1} = \beta_{\_REMISSION} * REMISSION + \beta_{\_REDUCTION} * REDUCTION + \beta_{\_DEPRESSION} * DEPRESSION + \beta_{\_MEMORY} * MEMORY + \beta_{\_AGGRESSION} * AGGRESSION$$

Where U is the utility of an AED.

The parameter estimates ($\beta_{\_REMISSION}$, $\beta_{\_REDUCTION}$, $\beta_{\_DEPRESSION}$, $\beta_{\_MEMORY}$, $\beta_{\_AGRESSION}$) measure marginal utility of a change in that outcome[9]. These β coefficients were used as preference weights that were multiplied by observed outcomes (REMISSION, REDUCTION, DEPRESSION, MEMORY, AGGRESSION).

Maximum acceptable risk:

The coefficients (β) from the regression were used to calculate the maximum acceptable risk (MAR) of an adverse event (AE) that respondents were willing to accept in exchange for a percentage point improvement in benefit (12-month remission).

The MAR was calculated by dividing the change in utility for a 1% improvement in remission by the change in utility for a 1% risk of AE, as follows:

$$MAR_{AE} = \beta_{\_REMISSION} / \beta_{\_AE}$$

This formula calculates the risk increase that would leave utility unchanged is AED efficacy is increased[iii].

The acceptability of a switch to the AED with the highest rate of remission, as observed in the clinical trial, was therefore assessed by calculating the patient-defined maximum acceptable risk associated with the corresponding improvement in 12-month remission.

The MAR for each AE was the incremental risk that could be offset by the increase in utility derived from better efficacy[34]. For the switch to be acceptable the following condition must be satisfied:

$$(MAR_{AE} * \Delta\%_{REMISSION}) \geq \Delta\%_{AE}$$

Where:

$$\Delta\%_{REMISSION} = (\%_{REMISSION\_ALTERNATIVE} - \%_{REMISSION\_CURRENT})$$

$$\Delta\%_{AE} = (\%_{AE\_ALTERNATIVE} - \%_{AE\_CURRENT})$$

Uncertainty Analysis

Confidence intervals (95%) were determined using 1000 bootstrap replications.

[a] Utility model based on Lancaster's Theory of Economic value [Utility = ∑preference-weighted outcomes], which contends that goods and services (or medicines in this case) can be described by their characteristics or attributes and that the utility yielded by a medicine is a function of its various attributes (Lancaster, 1966)[iv]. Attributes can be traded to maximise and maintain utility.

References

i. Hahn GJ, Shapiro SS. A Catalogue and Computer Programme for the Design and Analysis of Orthogonal Symmetric and Asymmetric Fractional Factorial Experiments. Technical information Series (66-Cl 65). 1966.

ii. Street D, Burgess L. The Construction of Optimal Stated Choice Experiments: Theory and Methods. London: Wiley, 2001.

iii. Johnson FR, Hauber BA, Poulos CM. A brief introduction to the use of stated-choice methods to measure preferences for treatment benefits and risks. RTI Press Publication No. RR-0009-0909. Research Triangle Park, NC: RTI International.

iv. Lancaster KJ. A new approach to consumer theory. The Journal of Political Economy, 1966;132-157.
